# Chromoblastomycosis in a renal transplant patient^⋆^

**DOI:** 10.1016/j.abd.2023.02.008

**Published:** 2023-09-23

**Authors:** Ingrid Rocha Meireles Holanda, Priscila Neri Lacerda, Carolina Nunhez da Silva, Rosangela Maria Pires de Camargo, Anna Carolina Miola, Silvio Alencar Marques

**Affiliations:** Infectious Diseases, Dermatology, Imaging Diagnosis and Radiotherapy, Faculty of Medicine, Universidade Estadual Paulista, Botucatu, SP, Brazil

Dear Editor,

Chromoblastomycosis is a subcutaneous mycosis caused by the traumatic implantation of dematiaceous fungi, primarily affecting the lower limbs of male rural workers in tropical and subtropical regions.^1^ It is rarely reported in immunosuppressed hosts, particularly in solid-organ transplant recipients or in association with neoplastic diseases.^2^, ^3^ This report describes a 63-year-old male construction worker, living in an urban area, who reported an injury in the right forearm after local trauma caused by a tree branch three years ago. Growth was slow and progressive, with mild local pruritus. The patient was hypertensive and had received a renal transplant five years before due to chronic kidney disease, probably secondary to the use of non-steroidal anti-inflammatory drugs and taking, used tacrolimus 2 mg/day, sirolimus 2 mg/day and prednisone 5 mg/day.

On examination, an infiltrated erythematous-squamous plaque measuring 2.5 × 3.0 cm including the scarring area, was observed (Fig. 1A). Dermoscopy, using a Heine device, model Delta 30, under polarized light, showed reddish-pink background, scales, reddish-orange ovoid structures interspersed with brown dots and extravascular red lacunnae in an area not covered by squamous crusts (Fig. 1B).

**Figure 1:**
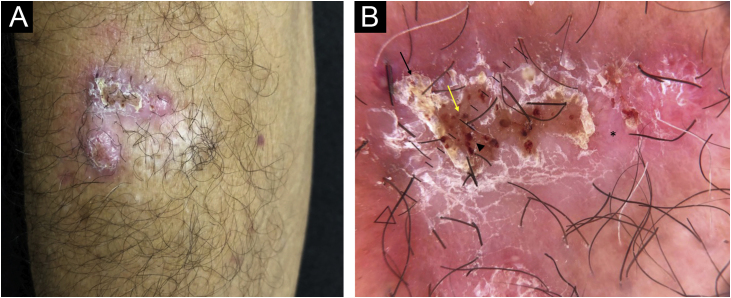
(A) Infiltrated plaque lesion, on the right forearm, showing surface covered by squamous crusts. (B) Dermoscopy under polarized light showed a reddish-pink background (asterisk), desquamation (black arrow), reddish-orange ovoid structures interspersed with brown dots (yellow arrow) and extravascular red lacunnae in an area not covered by squamous crusts (arrowhead)

Histopathology showed the presence of a dermal granulomatous inflammatory infiltrate, associated with neutrophilic microabscess and frequent round, brownish fungal structures, characteristic of sclerotic or muriform cells, some of them inside the cytoplasm of multinucleated giant cells (Fig. 2) presenting equatorial septation. Culture on Mycosel agar showed a greenish-black velvety colony with central elevation (Fig. 3A) and the microculture revealed *Cladosporium*-type sporulation with erect conidiophores, short chains, and two to three conidia at or near the apex, compatible with *Fonsecaea* spp. (Fig. 3B). Due to the COVID-19 pandemic, the patient was reassessed nine months after the initial appointment, reporting that the lesion was stable. He informed the replacement of sirolimus by mycophenolate mofetil during the period and underwent surgical excision, with a 5-mm margin. The patient returned for reavaliation two months after surgery with good healing and no signs of recurrence (Fig. 4), maintaining the scarred aspect at the time of the last reassessment, corresponding to the 9^th^ month after the surgical procedure.

**Figure 2:**
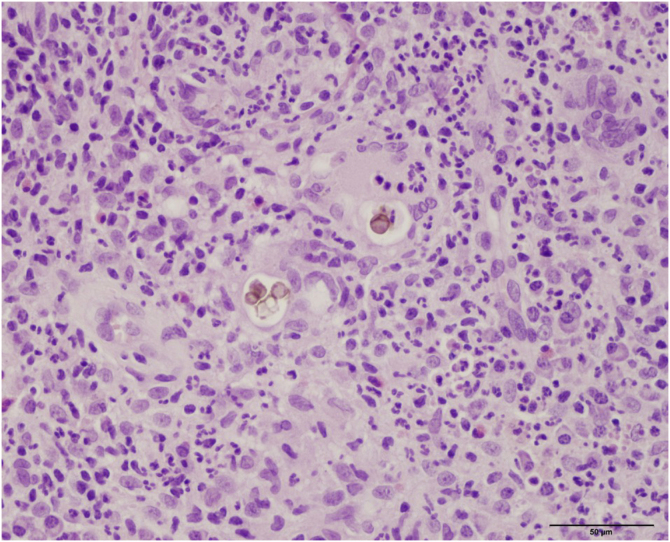
Dermal granulomatous inflammatory infiltrate, associated with neutrophilic abscess and the presence of brownish-colored fungi, muriform bodies, some of them inside the cytoplasm of multinucleated giant cells (Hematocylin & eosin 40X)

**Figure 3:**
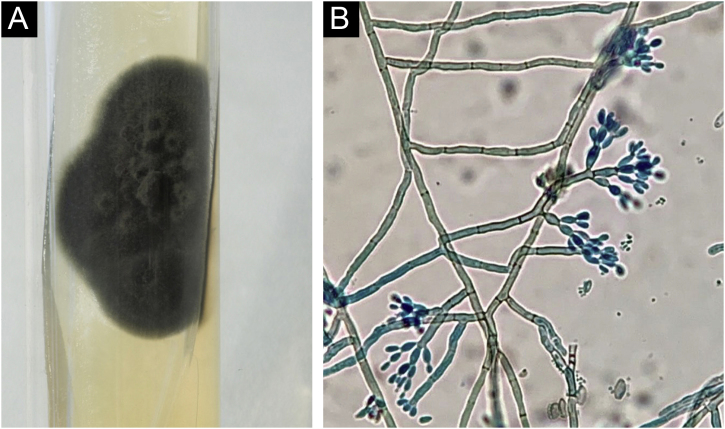
(A) Velvety greenish-black culture, with a central elevation. Mycosel agar. (B) Microculture showing the presence of *Cladosporium*-type sporulation with erect conidiophores, and short chains with two to three conidia. (Staining: cotton blue). *Fonsecaea* spp

**Figure 4:**
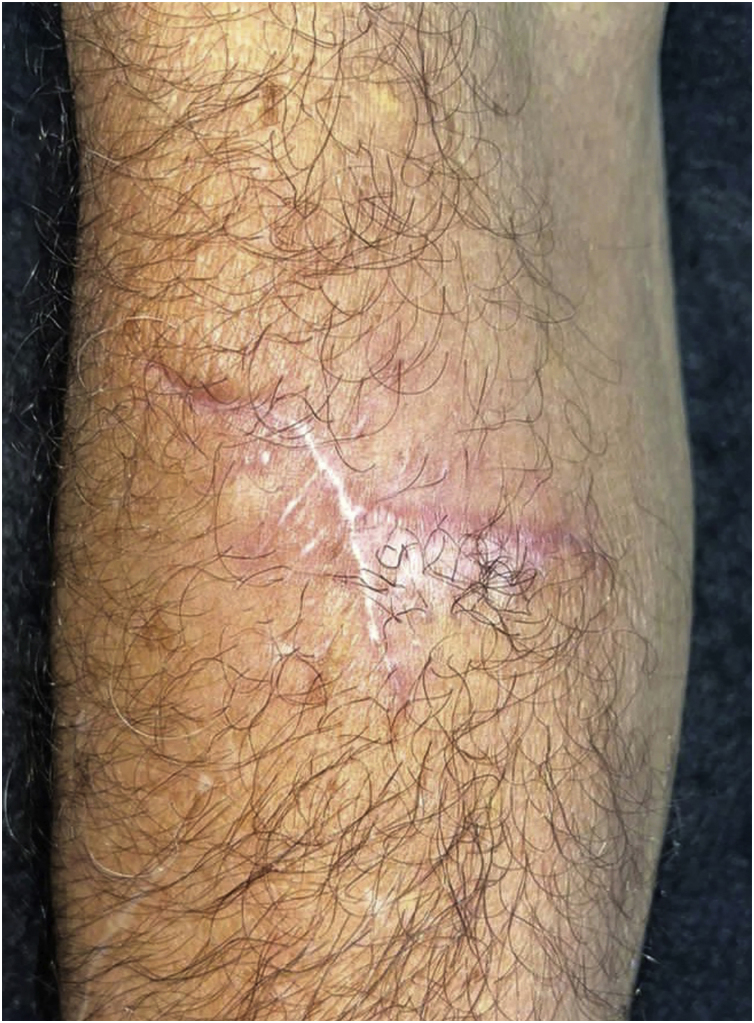
Late postoperative period, two months after excisional surgery

The countries with the highest prevalence of chromoblastomycosis are Brazil, Mexico, Venezuela, India, Australia, and China^2^ In Brazil, this mycosis occurs in most states, and the state of Pará presents the highest incidence.^1^ The lower limbs are the most affected areas, followed by the upper limbs, and males aged between 20‒60 years, with rural activity are the common denominator in 90% of cases.^1^, ^2^, ^3^, ^4^, ^5^ The most prevalent etiological agent is *Fonsecaea pedrosoi* (66%–96% of cases), followed by *Cladophialophora carrionii*, and *Phialophora verrucosa*.^2^, ^3^, ^4^, ^5^ The clinical manifestation of chromoblastomycosis is polymorphic and may present as a verrucous, nodular, tumor-like, cicatricial, or infiltrated plaque.^2^ The evolution of lesions associated with post-transplant immunosuppression does not differ, in most cases, from that observed in immunocompetent patients, i.e., slow, indolent growth.^2^, ^3^, ^4^, ^5^ It can be speculated that tacrolimus, due to its antifungal action, is also effective in delaying lesion progression.^6^

The presence of diseases or the use of associated immunosuppressive medications was described in only 0.2% of the reported cases, with solid-organ transplantation being the most common, followed by HIV infection, rheumatoid arthritis, systemic lupus erythematosus (SLE), bladder cancer, celiac disease, pernicious anemia and non-Hodgkin's lymphoma.^3^, ^4^, ^5^

Dermoscopy usually shows the presence of reddish-black dots that represent the transepidermal elimination of inflammatory cells, fungal elements, or hemorrhage. Yellow-orange ovoid structures on a pinkish-white background, polymorphic vessels, scales, and crusts may also be seen.^7^ The treatment of choice for chromoblastomycosis, whenever possible, is surgical removal, with or without clinical treatment or adjuvant physical treatments that can reduce the lesion diameter and allow surgical excision. Long-term postoperative follow-up is recommended, as recurrence is possible. Itraconazole 200–400 mg/day for a variable time, or in combination with terbinafine 500 mg/day are the most used antifungals. Posaconazole is an alternative treatment after failure of or intolerance to classical clinical treatment.^8^ Physical therapeutic methods such as thermotherapy, cryotherapy, laser therapy, and photodynamic therapy may be effective or useful as adjunct treatments.^2^

Chromoblastomycosis constitutes a diagnostic challenge in immunosuppressed patients, as it is rarely observed or reported in the literature. It is important to be aware of drug interactions between systemic antifungals and the immunosuppressive therapy being used, which may constitute an important limiting factor for treatment.

## Financial support

None declared.

## Authors’ contributions

Ingrid Rocha Meireles Holanda: Design and planning of the study; drafting and editing of the manuscript; collection, analysis, and interpretation of data; effective participation in research orientation; intellectual participation in the propaedeutic and/or therapeutic conduct of the studied cases; critical review of the literature; critical review of the manuscript; approval of the final version of the manuscript.

Priscila Neri Lacerda: Design and planning of the study; drafting and editing of the manuscript; collection, analysis, and interpretation of data; intellectual participation in the propaedeutic and/or therapeutic conduct of the studied cases; critical review of the literature; critical review of the manuscript.

Carolina Nunhez da Silva: Drafting and editing of the manuscript; collection, analysis, and interpretation of data; intellectual participation in the propaedeutic and/or therapeutic conduct of the studied cases; critical review of the literature; critical review of the manuscript.

Rosangela Maria Pires de Camargo: Drafting and editing of the manuscript; collection, analysis, and interpretation of data; intellectual participation in the propaedeutic and/or therapeutic conduct of the studied cases; critical review of the literature; critical review of the manuscript.

Anna Carolina Miola: Drafting and editing of the manuscript; collection, analysis, and interpretation of data; intellectual participation in the propaedeutic and/or therapeutic conduct of the studied cases; critical review of the literature; critical review of the manuscript.

Silvio Alencar Marques: Design and planning of the study; drafting and editing of the manuscript; effective participation in research orientation; intellectual participation in the propaedeutic and/or therapeutic conduct of the studied cases; critical review of the literature; critical review of the manuscript; approval of the final version of the manuscript.

## Conflicts of interest

None declared.
